# The Influence of Commercial Feed Supplemented with *Carnobacterium maltaromaticum* Environmental Probiotic Bacteria on the Rearing Parameters and Microbial Safety of Juvenile Rainbow Trout

**DOI:** 10.3390/ani12233321

**Published:** 2022-11-28

**Authors:** Iwona Gołaś, Jacek Arkadiusz Potorski

**Affiliations:** Department of Water Protection Engineering and Environmental Microbiology, University of Warmia and Mazury in Olsztyn, Prawocheńskiego 1, 10-720 Olsztyn, Poland

**Keywords:** juvenile rainbow trout, commercial feed, rearing parameters, apparent digestibility of nutrients, *C. maltaromaticum* environmental probiotic isolate, potentially pathogenic bacteria in the digestive tract and skin of the fish

## Abstract

**Simple Summary:**

Rainbow trout meat is characterized by a high nutritional value and microbial safety. The growing demand for trout meat has promoted the rapid development of modern aquaculture methods. Fish feeds are supplemented with various strains of probiotic bacteria to conform to the modern production requirements in aquaculture. Probiotic bacteria, including *Carnobacterium maltaromaticum*, are defined as live microorganisms that confer health benefits. The beneficial influence exerted by probiotics on fish growth and welfare is determined by the microbial species, its metabolic activity, and origin. However, commercial cultures of probiotic bacteria generally do not deliver the anticipated effects on the farming of fish and other aquatic organisms. In this study, fish feed was supplemented with a probiotic strain of *C. maltaromaticum* that naturally colonizes cold water in deep lake strata. Juvenile rainbow trout were fed commercial feed supplemented with the analyzed bacterial isolate. Feed supplementation significantly increased the fish biomass, improved the apparent digestibility of feed and nutrients, and contributed to a several–fold decrease in the counts of potentially pathogenic bacteria in the feed, digestive tract contents, and the skin of fish. The results of this study indicate that the *C. maltaromaticum* environmental strain is a promising probiotic for rearing juvenile rainbow trout in aquaculture.

**Abstract:**

The aim of this study was to determine the effect of commercial feed (CF) supplemented with 0.1% of the *Carnobacterium maltaromaticum* environmental probiotic strain on the rearing parameters, apparent nutrient digestibility, and microbial safety of juvenile rainbow trout (*Oncorhynchus mykiss*). The fish were fed CF (control group, CG) and experimental feed (EF) supplemented with 0.1% of *C. maltaromaticum* (experimental group, EG) for 56 days. The final body weight and total body length of the fish were measured. The growth rate, condition factor, feed conversion ratio, viscerosomatic and hepatosomatic indices, and apparent digestibility coefficients of protein (PAD), lipids (LAD), ash (AAD), and nitrogen-free extract (NFEAD) were calculated. The total viable counts of *C. maltaromaticum* bacteria, mesophilic bacteria, hemolytic mesophilic bacteria, *Pseudomonas fluorescens*, *Aeromonas hydrophila*, *Staphylococcus* sp., and sulfite-reducing anaerobic spore-forming *Clostridium* sp. were determined in digestive tract contents and the skin of fish. Feed supplementation with *C. maltaromaticum* significantly affected most rearing parameters, as well as the PAD, LAD, AAD and NFE values, and bacterial counts. The principal component analysis (PCA) revealed significant positive correlations (*p* < 0.05) between fish growth rates, PAD and LAD values vs. *C. maltaromaticum* counts in the EF and in the digestive tract contents of the fish.

## 1. Introduction

According to FAO data [1], global trout production reached 939.878 tonnes in 2019 and has been increasing since 2015 (21% increase in volume between 2015 and 2019). The rainbow trout was the main species of farmed fish that accounted for 97% of the total production volume in 2019. In the European Union countries, rainbow trout is farmed for human consumption and for re-stocking water bodies for recreational angling [2]. Around 56% of young specimens are farmed in recirculating aquaculture systems (RAS) [3]. In these systems, fish are bred and raised under controlled conditions, and the physicochemical and microbiological parameters of the aquatic environment are monitored in each stage of the production process; therefore, the presence of environmental microorganisms is limited to feed microbiota [4]. The type of diet (including the content of protein, lipids, ash, nitrogen-free extract (NFE), vitamins, and micronutrients) affects feed conversion efficiency (feed conversion ratio (FCR), apparent nutrient digestibility) in different fish species and across different developmental stages. Fish diets also significantly influence fish rearing parameters, including biomass weight and length gain, specific growth rate, daily growth rate, condition factor, as well as embryogenesis, ovulation, immunity, stress responses, and adaptive mechanisms in fish [5,6,7]. In aquaculture, the effectiveness of juvenile fish rearing is influenced not only by the composition of feed, but also by its microbiological quality. Feed microbiota colonize the gastrointestinal tract of fish and facilitate nutrient digestion [8,9,10,11]. However, feed contaminated with potentially pathogenic microorganisms poses a health threat to fish. Juvenile fish are particularly susceptible to pathogenic microbiota. High counts of pathogenic bacterial strains such as *P. fluorescens*, *A. hydrophila*, *Staphylococcus* spp., and *Clostridium* spp. can compromise fish health and decrease survival rates [12]. Virulent microbiota cause diseases that disrupt the digestive processes, decrease nutrient assimilation and, consequently, reduce fish biomass gain. In addition, fish and fish feces evacuated to the aquatic environment can act as secondary sources of pathogenic microorganisms in RAS.

In aquaculture, potentially pathogenic and pathogenic microorganisms are difficult to eliminate because only a small number of pharmacological products have been approved for use in RAS. Chemotherapeutic agents exert a negative impact on other components of aquaculture, the surrounding environment, and organisms colonizing that environment, which prompts the search for new methods to protect the health of farmed fish [13,14].

Various species of probiotic microorganisms, including *Lactobacillus* spp., *Lactococcus* spp., *Enterococcus* spp., *Carnobacterium* spp., *Streptococcus* spp., *Bacillus* spp., *Aeromonas* spp., *Vibrio* spp., *Enterobacter* spp., and *Pseudomonas* spp., play an important role in closed aquaculture systems [15,16,17]. The extent to which probiotics confer health benefits on the host is determined by the microbial species, its metabolic activity, and origin. There is considerable evidence to suggest that probiotic bacteria enhance fish health, increase body weight and length gains, enhance the activity of digestive enzymes, eliminate pathogenic bacteria, and stimulate the immune system [17,18,19,20,21,22,23]. However, little is known about the environmental impact or the side effects of probiotics in animal rearing. Probiotic preparations applied in animal (veterinary) husbandry are used in commercial rearing, and they are introduced directly to aquaculture without considering their future impact [24]. For this reason, the properties of probiotic bacteria and their influence on the water environment and other aquatic organisms should be investigated before these strains are introduced to fish farms. Probiotic strains isolated from fish, other aquatic organisms, or water seem to be the safest option for fish farms. Due to their origin, such strains grow and develop rapidly in RAS, and their metabolic activity and antimicrobial properties are enhanced in aquaculture. This group includes *Carnobacterium maltaromaticum*, which is one of the most metabolically active probiotic bacteria [25,26]. This bacterial species is tolerant to changes in environmental conditions (temperature, pH, salinity), and it easily adapts to various animal habitats. Therefore, the growth and activity of *C. maltaromaticum* is not compromised by the presence of xenobiotics such as antibiotics and disinfectants, or diverse microbial populations that constitute natural and potentially pathogenic fish microbiota [25,27,28]. A limited number of studies have confirmed that *C. maltaromaticum* is a promising probiotic for aquaculture [25,26,29].

In view of the far-reaching goals of modern aquaculture, the aim of this study was to determine the influence of commercial feed (CF) supplementation with 0.1% of the *C. maltaromaticum* environmental probiotic strain on the production of juvenile rainbow trout (*Oncorhynchus mykiss*) based on: (i) fish rearing parameters, (ii) apparent nutrient digestibility, and (iii) changes in the quantitative and qualitative composition of potentially pathogenic microbiota in the digestive tract contents and the skin of fish.

## 2. Materials and Methods

### 2.1. Fish and Rearing Conditions

**Figure 1:**
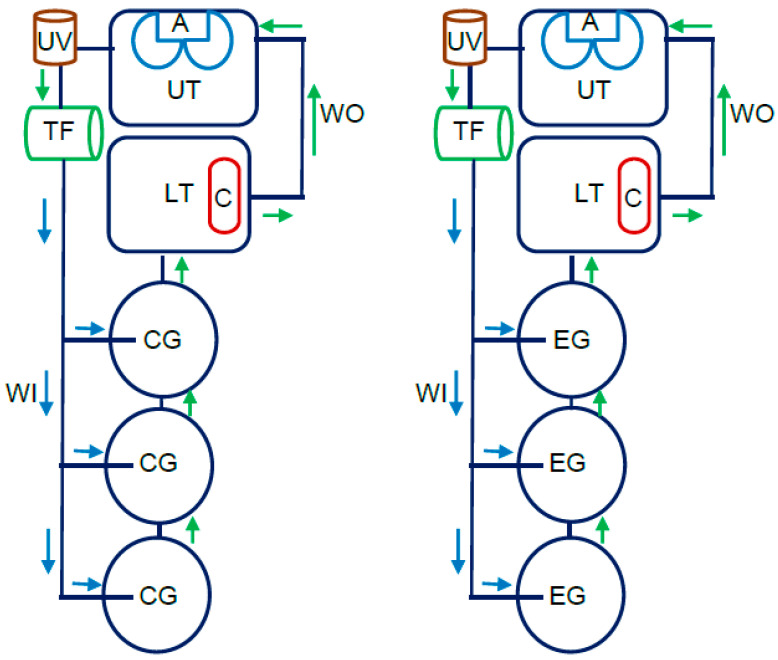
Diagram of juvenile rainbow trout rearing in a recirculating aquaculture system (RAS). Abbreviations: CG—rearing tanks stocked with control group fish; EG—rearing tanks stocked with experimental group fish; LT—lower tank, C—coil heater; A—aerators, UV—UV lamp; TF—trickling filter; WI—water inflow; WO—water outflow.

This study involved juvenile rainbow trout (*Oncorhynchus mykiss*) that were experimentally reared in RAS in the Department of Ichthyology (Center of Aquaculture and Environmental Engineering) of the University of Warmia and Mazury in Olsztyn (Poland). Two isolated RAS were set up for the experiment (Figure 1). Each RAS comprised three rearing tanks (with a volume of 250 L each) filled with mains water at a flow rate of 4 L min^−1^ [30]. Each day, 10% of total water volume was recirculated in the RAS. Juvenile rainbow trout for the experiment were obtained from Wodna Farma Ltd. in Wirwajdy (Poland), and they had a mean initial body weight (BW_i_) of 44.20 ± 7.73 g and a mean initial body length (BL_i_) of 15.5 ± 0.1 cm [31].

A total of 6 rearing tanks were randomly stocked with juvenile rainbow trout (180 individuals) (2 groups with 3 replicates each: 3 control groups and 3 experimental groups). The fish were acclimated for 14 days according to NRC recommendations [32]. During the acclimation period, juveniles were fed commercial feed (CF), Aller Gold (Aller Aqua, Denmark), which was administered at 1.3% of the fish biomass. The optimal water temperature for rainbow trout (16.1 ± 0.20 °C) was maintained during the 8-week rearing experiment. The following water parameters were measured daily before fish feeding: oxygen content (±0.01 mg O_2_ L^−1^), water pH (±0.01), content of total ammonia nitrogen (TAN = NH^3+^–N + NH^4+^–N) (±0.01 mg TAN L^−1^), nitrite nitrogen NO_2_–N (±0.01 mg NO_2_–N L^−1^), and nitrate nitrogen NO_3_-N (±0.01 mg NO_3_–N L^−1^). The oxygen content, TAN, NO_2_–N, NO_3_–N, and water pH at tank outflow were as follows: 8.05 ± 0.25 mg O_2_ L^−1^, 0.05 ± 0.02 mg TAN L^−1^, 0.020 ± 0.002 mg NO_2_–N L^−1^ and 0.20 ± 0.02 mg NO_3_–N L^−1^, and 7.20 ± 0.10. During the entire experiment, the physical and chemical parameters of tank water were maintained at the optimal levels recommended for rainbow trout [33]. The fish were exposed to a 8L: 16D photoperiod, and light intensity at tank surface was 40 to 80 lux [34]. After the experiment, the fish were euthanized with a solution of 300 mg tricaine methanesulfonate (MS–222) L^−1^ (FINQUEL, Redmond, WA, USA) [35].

### 2.2. Feed and Feeding

During the experiment, juvenile rainbow trout were fed Aller Gold CF (Aller Aqua, Denmark) with pellet diameter of 3.0 mm. According to the manufacturer’s specifications, the feed had the following composition: 44.0% crude protein, 28.0% crude fat, 14.0% NFE, 8.0% crude ash, and 1.9% crude fiber. Gross and digestible energy was 23.5 MJ kg^−1^ and 21.3 MJ kg^−1^, respectively. Control group (CG) fish received CF. Experimental group (EG) fish received experimental feed (EF) comprising Aller Gold feed supplemented with 0.1% (6.5 × 10^8^ cfu g^−1^) of the *C. maltaromaticum* environmental probiotic strain. Control and experimental feeds contained 1% of chromic oxide (Chempur, Piekary Śląskie, Poland) to determine nutrient digestibility. The feeds were extruded in the TS-4D extruder (Metalchem, Poland) with a 2.0 mm die. The extrusion parameters were as follows: temperature at the conditioner outlet (°C): 120, temperature in the second segment (°C): 110; endplate temperature (°C): 120, endplate pressure (bar): 15, screw speed (rpm): 60, cutter speed (rpm): 20.

The EF was supplemented with an environmental probiotic strain of *C. maltaromaticum* isolated from water samples collected from the benthic zone of Lake Legińskie (at a depth of 34 m) in north-eastern Poland (N = 53°58′51″ N and E = 21°8′4″). The strain had been isolated during a previous study conducted by the Department of Environmental Microbiology of the University of Warmia and Mazury in Olsztyn (Poland). The isolate was identified by 16S rDNA (recombinant DNA) sequencing with the BigDye Terminator v3.1 kit in the ABI 3730xl genetic analyzer (Applied Biosystems, Foster City, CA, USA). In addition, 16S rDNA genes were sequenced by PCR with the use of 27F (5′–AGAGTTTGATCATTGGCTCAG–3′) and 1492R (5′-GGTACC-TTGTTACGACTT-3′) primers according to the method described by Gillan [36]. The DNA sequences were identified with the BLAST program available on the website of the National Center for Biotechnology Information (http://blast.ncbi.nlm.nih.gov/Blast.cgi, accessed on 20 March 2016) [37]. The results of 16S rDNA sequencing are presented in Table S1 (Supplementary Materials). The probiotic potential, metabolic activity, and applicability of *C. maltaromaticum* as a supplement for fish feed had been examined previously [31,38,39].

Fresh 24 h cultures of *C. maltaromaticum* on tryptone soy agar (TSA; Oxoid, Basingstoke, UK) with the addition of 3% yeast extract and 1.5% (*w*/*v*) NaCl [25] were lyophilized in the ALPHA 2–4 LDplus freeze dryer (Martin Christ Gefriertrocknungsanlagen GmbH, Osterode am Harz, Germany). A lyophilized probiotic strain of *C. maltaromaticum* (6.5 × 10^8^ cfu g^−1^ EF) was added to the mixture of fish oil and soybean oil (5% each). Next, the probiotic oil suspension was added to the EF sample. The oil mixture was pumped into the feed at 0.9 MPa for 5 min with the use of a vacuum pump. Control and experimental feeds were stored in a refrigerator (Whirlpool, Benton Harbor, MI USA) at a temperature of 4–6 °C during the entire experiment [31]. Feed was administered every 12 h by automatic feeders (FIAP, Inverness, UK). The CG and EG fish received feed in the daily amount of 1.3% of their biomass [34].

### 2.3. Fish Measurements and Sampling

Individual fish were weighed and measured (body weight, BW ± 0.01 g, total length, BL ± 0.1 cm) at the beginning and end of the experiment. Fish biomass was determined weekly in each tank by weighing the entire stock (±1.0 g). Livers and digestive tracts were weighed at the end of the experiment, immediately after euthanasia. The collected data were used to calculate the following parameters:

- specific growth rate in percent per day:

SGR (% day^−1^) = 100 × (ln BW_f_ − ln BW_i_) × T^−1^ (1)

- daily growth rate in grams per day:

DGR (g day^−1^) = (BW_i_ − BW_f_) × T^−1^ (2)

- condition factor:

K_f_ (%) = 100 × (BW × BL^−3^) (3)

- coefficient of variability of body weight on the first (CVBW_i_) and last (CVBW_f_) day of the experiment:

CVBW (%) = 100 (SD × BW^−1^) (4)

feed conversion ratio: FCR = TFI × (FB − IB)(5)

viscerosomatic index: VSI (%) = 100 × (Wv × BW_f_^−1^) (6)

hepatosomatic index: HIS (%) = 100 × (Wl × BW_f_^−1^) (7)

Parameters BW_i_ and BW_f_ denote the initial and final body weights (g), respectively; T is rearing time (days); BL is body length (cm); SD is standard deviation of the average body weight; FB and IB are the final and initial stock biomass (g), respectively; TFI is total feed intake (g); Wv is the weight of viscera (g); Wl is liver weight (g).

### 2.4. Apparent Nutrient Digestibility

To determine the apparent digestibility coefficients of protein, lipids, ash, and nitrogen-free extract (NFE), 5 fish were collected from each tank (15 fish from each dietary treatment) on the first and last day of the experiment. The fish were anesthetized in an anesthetic solution (300 mg L^−1^), decapitated, and the hindguts were excised. The proximate analysis of feeds and feces (crude protein, crude fat, and crude ash) was performed according to standard AOAC methods [40]. Total protein content was determined using Kjeldahl’s method, and crude fat content using Soxhlet’s method. Nitrogen-free extract was calculated according to Shearer [41] with the following formula:

NFE (%) = 100 − [(moisture (%) + protein (%) + lipids (%) + ash (%) + fiber (%)]. (8)

The content of chromic oxide in diets and feces was determined according to the method of Furukawa and Tsukahara [42].

The apparent digestibility coefficients of protein, lipids, and ash was calculated according Maynard et al. [43] as:

PAD, LAD, and AAD (%) = 100 − [(Cr_2_O_3_ in feed (%)) × (Cr_2_O_3_ in feces (%))^−1^ × (protein or lipids or ash in feces (%)) × (protein or lipids or ash in feces and in feed (%))^−1^] × 100. (9)

### 2.5. Microbiological Analyses

Fifteen fish randomly sampled from CG and EG on the first and last day of each dietary treatment were subjected to microbiological analyses. Samples of digestive tract (1 g) contents and skin (1 g) were collected aseptically from anesthetized fish (in 300 mg of MS–222 solution L^−1^). Samples of feed: CF and EF (10 g each) were collected aseptically each week and analyzed. A total of 30 representative samples of digestive tract contents and skin from each dietary treatment, and 7 samples of each feed (CF and EF) were subjected to microbiological analyses.

Aliquots of 1 g fish samples (digestive tract contents, skin) and 10 g feed samples were homogenized with 9 mL and 90 mL of sterile phosphate-buffered saline (PBS), respectively. Serial dilutions with PBS were prepared to reduce bacterial density. Subsequently, 0.1 mL of every homogenized solution was subjected to microbiological analysis. The results of microbiological assays are presented in Table 1. All analyses were performed according to Polish Standards [44,45]. The counts of the analyzed bacterial groups, genera, and bacteria were determined with the use of previously described methods [39,46,47]. Mean microbial counts in the same sample of digestive tract contents, skin, and two fish feeds (CF and EF) were determined in triplicate. Total microbial counts were expressed in cfu g^−1^ of digestive tract contents, skin, and feed.

### 2.6. Statistical Analysis

Statistical analyses were carried out using the Statistica 13.3 software package (TIBCO Software Inc., Palo Alto, CA, USA). The Shapiro–Wilk test was applied to test the normality of data distribution. The differences (*p* < 0.05) in rearing parameters and apparent nutrient digestibility were analyzed using the Mann–Whitney U test. All percentage data were transformed with the square arcsine transformation before statistical analysis.

The significance of differences in bacterial counts between CG and EG were determined using one-way analysis of variance (ANOVA). The correlations between *C. maltaromaticum* counts and the analyzed bacterial groups in fish and feed samples were determined using Spearman’s nonparametric rank correlation test (*p* < 0.05).

The presence of relationships between *C. maltaromaticum* counts in fish and feed samples (CF, EF) and fish rearing parameters was determined by principal component analysis (PCA) with supplementary variables using CANOCO 5.0 software [48]. All variables were standardized to zero mean and unit variance before PCA.

## 3. Results

### 3.1. Fish Growth Rate, Apparent Digestibility Coefficients of Protein, Lipids, Ash, and NFE

Significant differences (*p* < 0.05) in most growth parameters were observed between rainbow trout fed with the CF and EF (Table 2). Fish receiving the EF were characterized by significantly higher values of BW_f_, B_f,_ BW_g_, and DGR (by 14.44 ± 6.52%, 14.44 ± 6.52% 24.78 ± 8.14%, and 25.34 ± 4.23, respectively) than fish fed with the CF. Feed supplemented with 0.1% of the *C. maltaromaticum* environmental probiotic strain significantly (*p* < 0.05) decreased the intra-group coefficient of variation of body weight (CVBW) in both the CG and EG. As a result, the mean values of CVWB were determined at 1.34 ± 0.16 in the CG and 1.20 ± 0.13 in the EG, and the difference was statistically significant (*p* < 0.05). The addition of the *C. maltaromaticum* probiotic isolate to the Aller Gold feed significantly (*p* < 0.05) influenced the FCR, specific growth rate (SGR), viscerosomatic index (VSI), and the final condition factor (K_f_). In fish fed with the EF, the values of SGR, VSI, and K_f_ increased (by 13.77 ± 0.04%, 9.00 ± 1.89%, and 4.14 ± 0.53%, respectively), whereas FCR decreased (by 16.14 ± 0.05%) in comparison with fish fed with the CF.

The addition of the *C. maltaromaticum* probiotic isolate to the fish diets also significantly (*p* < 0.05) influenced the apparent digestibility coefficients of protein (PAD), lipid (LAD), ash (AAD), and NFE (ADNFE) (Table 2). In the EG fish receiving the Aller Gold feed with 0.1% addition of the probiotic bacteria, the values of the digestibility coefficients increased by 3.29 ± 0.31%, 1.00 ± 0.09%, 1.01 ± 0.07, and 5.13 ± 0.35, respectively, relative to the CG fish whose diets were not supplemented (Table 2).

### 3.2. Counts of Potentially Pathogenic Microbiota in the Digestive Tract and the Skin of Fish

**Figure 2:**
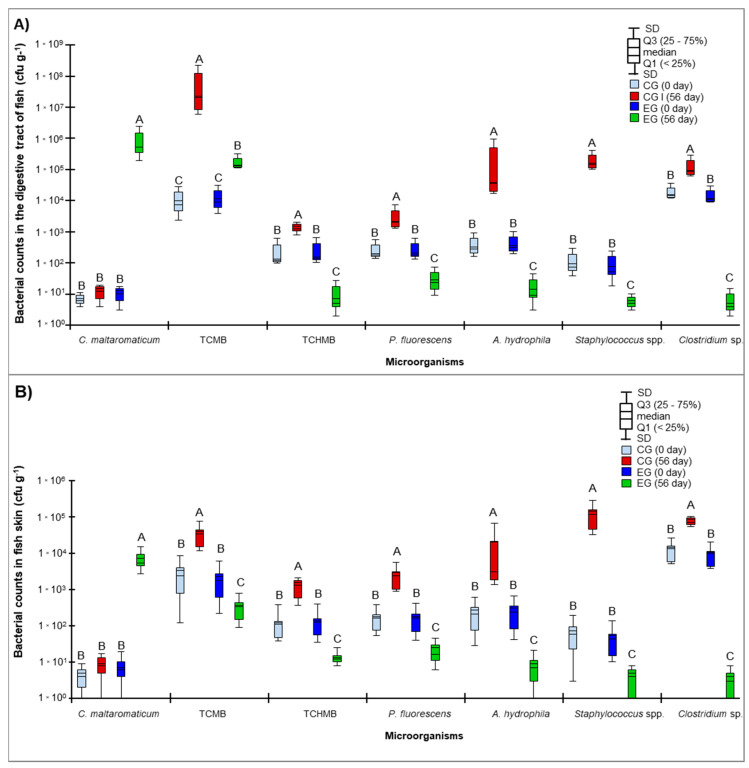
Bacterial counts (third quartile, median, standard deviation) (cfu g^−1^) in the digestive tract (**A**) and skin (**B**) of the control (CG) and experimental groups (EG) of juvenile rainbow trout administered Aller Gold commercial feed and the same feed supplemented with 0.1% of the *Carnobacterium maltaromaticum* environmental probiotic strain, respectively. The numbers marked with different superscript letters differ significantly (ANOVA; *p* < 0.05; *n* = 30). Denotations: 0 day—first day of experiment, 56 day—last day of experiment, TCMB—total counts of mesophilic bacteria; TCHMB—total counts of hemolytic mesophilic bacteria.

Microbial counts in the digestive tract and skin samples ranged from 10^0^ to 10^8^ cfu g^−1^, depending on the sampling date (first or last day of the experiment) and the fish group (control or experimental). The EF containing 0.1% of the probiotic isolate affected the counts of potentially pathogenic bacteria in both the digestive tract and skin samples (Figure 2A,B). The counts of hemolytic bacteria (TCHMB), *P. fluorescens*, *A. hydrophila*, *Staphylococcus* spp., and *Clostridium* spp. were significantly reduced (ANOVA; *p* < 0.05) in the digestive tract and the skin of fish fed with the EF in comparison with juvenile rainbow trout fed with the CF (without 0.1% addition of *C. maltaromaticum*). On the last day of the experiment (day 56), *C. maltaromaticum* and TCMB counts in the digestive tract and the skin of the EG fish increased significantly (ANOVA; *p* < 0.05) relative to the first day of the experiment (day 0) (Figure 2A,B).

An analysis of the quantitative and qualitative composition of microbiota in the CF and EF revealed significant differences (ANOVA; *p* < 0.05; *n* = 14) in the microbial counts (4 to 6 orders of magnitude) between the examined feeds (Table 3).

The statistical analysis confirmed that the 0.1% addition of *C. maltaromaticum* (6.5 × 10^8^ cfu g^−1^) to the EF influenced the microbial counts in the digestive tract of the EG fish. The correlation analysis revealed significant positive correlations (*p* < 0.001–*p* < 0.05) between the probiotic isolate counts in the EF and in the digestive tract and skin samples (Table 4). The counts of *C. maltaromaticum* in the EF and the samples of the digestive tract and skin were negatively correlated (*p* < 0.001–*p* < 0.05) with the counts of the most potentially pathogenic bacteria in fish.

### 3.3. Correlations between Feed Rearing Parameters, the Apparent Digestibility Coefficients of Crude Protein, Crude Fat, Crude Ash, Nfe, and Digestive Tract Microbiota

**Figure 3:**
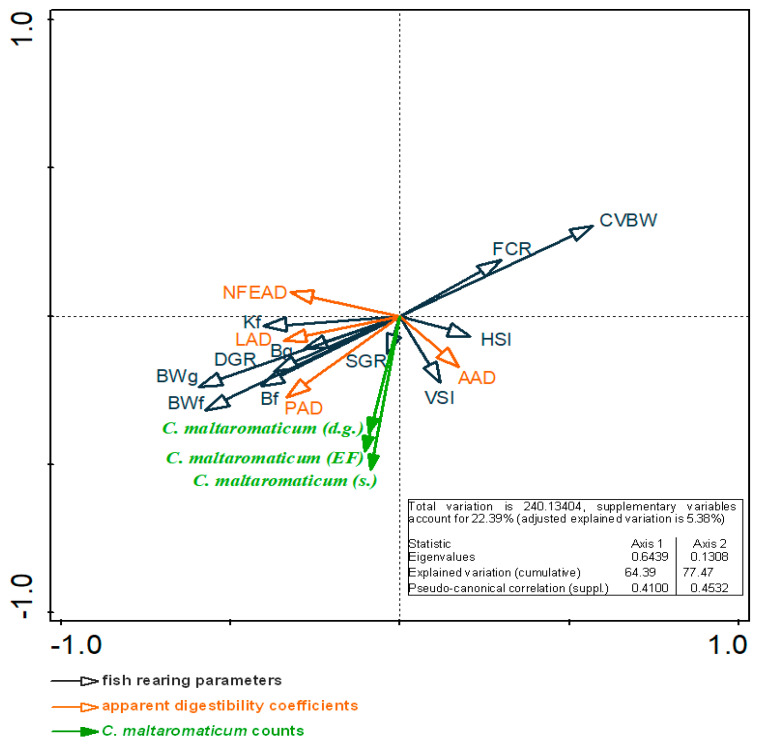
Principal component analysis (PCA) biplot of the correlations (*p* < 0.05) between *C. maltaromaticum* counts in the digestive tract (d.g.) and skin (s.) of juvenile rainbow trout vs. the experimental feed (Aller Gold supplemented with 0.1% of the *Carnobacterium maltaromaticum* environmental probiotic strain), rearing parameters (BW_g_—body weight gain, BW_f_—final body weight, B_f_—final biomass, B_g_—biomass gain, SGR—specific growth rate, K_f_—final condition factor, VSI—viscerosomatic index, DGR—daily growth rate, CVBW—coefficient of variation of body weight, FCR—feed conversion ratio, HIS—hepatosomatic index), and the apparent digestibility coefficients of crude protein (PAD), crude fat (LAD), crude ash (AAD), and nitrogen-free extract (NFEAD).

The PCA confirmed that the 0.1% addition of *C. maltaromaticum* to the EF had a positive effect on the fish rearing parameters and apparent nutrient digestibility (PAD, LAD, AAD, NFEAD). *Carnobacterium maltaromaticum* counts in the digestive tract contents were bound by significant positive correlations (*p* < 0.05; *n* = 45) with the values of B_g_, BW_g,_ BW_f_, B_f,_ DGR, SGR, and K_f_. The values of FCR and CVBW were negatively correlated (*p* < 0.05) with *C. maltaromaticum* counts in the digestive tract contents and the EF (Figure 3). The apparent digestibility analysis revealed that the *C. maltaromaticum* counts in the digestive tract contents were bound by a significant positive correlation (*p* < 0.05; *n* = 45) with PAD and LAD values (Figure 3).

## 4. Discussion

This study demonstrated that the supplementation of the CF with 0.1% of the *C. maltaromaticum* environmental probiotic strain significantly improved the rearing parameters of the juvenile rainbow trout in RAS. In the EG fish, the probiotic supplement induced a significant increase (Mann–Whitney U test; *p* < 0.05) in final body weight (BW_f_), body weight gain (BW_g_), daily growth rate (DGR), specific growth rate (SGR), condition factor (K_f_), and the viscerosomatic index (VSI) in comparison with the CG fish fed with the CF without supplementation. The EF significantly decreased the FCR. The PCA confirmed the presence of significant correlations between fish rearing parameters and *C. maltaromaticum* counts. In the present study, the supplementation of fish feed with the C. *maltaromaticum* environmental probiotic strain had no significant impact on the coefficient of variation of the final body weight (CVBW). The CVBW decreased in the EG on successive days of the experiment, which could suggest that rainbow trout do not exhibit strong stock domination or hierarchy [49].

Higher values of DGR, SGR, and FCR in fish fed with the EF validate the hypotheses postulating that the optimization of the ingredient composition of fish diets and feed rations maximizes feed utilization for growth and other activities, and improves fish welfare [50,51,52,53,54]. The presence of probiotic microorganisms in fish feed improves rearing parameters, and their beneficial influence is determined by the type of isolate, the applied dose, the fish species, the stage of development, and the type of feed [55]. The results of the present study confirm these observations. The values of BW_g_, DRG, and SGR increased (by approx. 25%, 26%, and 14%, respectively), whereas the FCR decreased (by approx. 17%) in the EG fish receiving feed supplemented with 0.1% of *C. maltaromaticum*. Merrifield et al. [21] reported an increase in the SGR values (from 2.01 to 2.15% day^−1^) and a decrease in the FCR values (from 0.98 to 0.93) in an experiment investigating the effect of feed supplemented with probiotics **(***Bacillus subtilis*, *B. licheniformis*, and *Enterococcus faecium*) on rainbow trout. In turn, Bagheri et al. [56] found that feed containing *B*. *licheniformis* and *B. subtilis* improved the FCR (from 1.00 to 0.85), DGR (from 3.30 to 3.80 g day^−1^), and modulated gut microbiota in rainbow trout fry. In a study by Mohapatra et al. [22], the FCR values decreased by around 35% in *Labeo rohita* fry receiving feed with the addition of *B. subtilis*, *Lactococcus lactis*, and *Saccharomyces cerevisiae* in comparison with fish whose diets were not supplemented. A rearing experiment involving Songpu mirror carp and catfish (*Clarias* spp.) demonstrated that a *B. megaterium*-coated diet increased the weight gains (BW_g_) and SGR values, and reduced the FCR, which suggests that these strains can promote fish growth [57,58].

The supplementation of the CF with 0.1% of the *C. maltaromaticum* environmental probiotic strain significantly improved apparent nutrient digestibility. The examined probiotic isolate significantly increased (*p* < 0.05) the apparent digestibility of crude protein (PAD), crude fat (LAD), and NFE (NFEAD) in the EF (by more than 3%, 1%, and 5%, respectively) relative to the CF. The PCA confirmed that the probiotic isolate improved the apparent nutrient digestibility, and it revealed significant correlations (PCA; *p* < 0.05) between the apparent digestibility coefficients of protein and fat vs. *C. maltaromaticum* counts in the Aller Gold feed. Similar results were reported by Sahandi et al. [59], who observed that feed supplementation with *Bifidobacterium* spp. bacteria increased the apparent digestibility coefficients of crude protein and crude fat (from 98.00% to 98.50%, and from 98.50% to 99.50%, respectively). In a rearing experiment involving *Labeo rohita* fingerlings (6.0 ± 0.06 g), Mohapatra et al. [22] found that a diet supplemented with *Bacillus subtilis*, *Lactococcus lactis*, and *Saccharomyces cerevisiae* probiotic isolates improved growth, protein efficiency ratio, nutrient retention, and digestibility. In the present study, the supplementation of the CF with 0.1% of *C. maltaromaticum* improved the EF conversion by stimulating nutrient digestibility [23,60,61]. Nutrient digestibility can be improved by including the enzymes produced by feed probiotic bacteria in the pool of digestive enzymes in the gastrointestinal tract of fish [62,63]. An increase in the activity of digestive enzymes (amylase, lipase, protease, trypsin) and growth factors (vitamins, fatty acids, amino acids), which are produced by gut microbiota, improves nutrient digestibility and absorption. Improved digestion is associated with better feed conversion [22,23,64,65], and it delivers indirect environmental benefits by reducing the volume of organic waste and decreasing the concentration of biogenic compounds in water, which is a particularly important consideration in RAS [66,67,68].

The results of the microbiological analyses revealed that *C. maltaromaticum* bacteria (added to the EF at a concentration of 6.5 × 10^8^ cfu g^−1^) significantly influenced microbial counts in the digestive tract and skin samples and in feed supplemented with this probiotic isolate. Significant positive relationships (*p* < 0.05) were noted between *C. maltaromaticum* counts in the EF and in samples of the digestive tract contents and skin. The concentrations of the probiotic isolate in the EF and in samples of the digestive tract contents and skin were negatively correlated (*p* < 0.05) with the counts of hemolytic bacteria (TCHMB), *P. fluorescens*, *A. hydrophila*, *Staphylococcus* spp., and *Clostridium* spp. At the end of the experiment, the counts of potentially pathogenic bacteria (excluding TCMB) in the digestive tract contents and skin of the EG fish decreased significantly (ANOVA; *p* < 0.05) relative to the CG fish. A decreasing trend (several orders of magnitude) in the total counts of potentially pathogenic bacteria was also noted in the EF supplemented with 0.1% of *C. maltaromaticum* relative to the CF. It should be noted that under controlled farming conditions in RAS, feed is the main source of biogenic compounds and microorganisms, and its microbiological quality directly affects the qualitative and quantitative composition of the gut microbiota in fish [18,19,57,69]. In turn, feed microorganisms indirectly influence the microbiota colonizing the water, skin, gills, eyes, and other anatomical structures in the fish [47,70]. Kim and Austin [71] and Zhang et al. [72] also reported that the *C. maltaromaticum* environmental probiotic strain exerted antagonistic effects against *Aeromonas salmonicida*, *A. hydrophila*, *Streptococcus iniae*, *Vibrio anguillarum*, *Serratia* spp., *Pseudomonas* spp., and *Bacillus* spp. bacteria. The counts of *V. anguillarum A. salmonicida V. ordalii*, and *Y. ruckeri* were also considerably reduced in the intestinal contents and skin mucus of salmonids when the *C. maltaromaticum* isolate was introduced to aquaculture [73,74]. Fish skin mucus is a biological reservoir of various immune molecules (lysozyme, proteins, immunoglobulins, enzymes, lectins, etc.), and it is a defense barrier against foreign particles, pathogens, and toxins [75]. Probiotics stimulate goblet cells in the epidermis by producing nutrients such as short-chain fatty acids, vitamins, and minerals, which enhance the secretion of antibacterial agents. Similar observations were made by Farsani et al. [76], who found that diets supplemented with *L. acidophilus* and *B. bifidum* significantly increased lysozyme levels in fish skin mucus.

According to the literature [77,78], *C. maltaromaticum* can be a fish pathogen. In most described instances, *C. maltaromaticum* exerted pathological effects only on severely stressed fish, for example during spawning. In the present study, feed supplementation with this probiotic isolate did not compromise fish welfare parameters (swimming behavior, feeding behavior, skin and fin damage) during experimental rearing of juveniles in RAS. In this study, the *C. maltaromaticum* environmental probiotic strain exerted antagonistic effects against a broad range of potentially pathogenic bacteria, including TCHMB, *P. fluorescens*, *A. hydrophila*, *Streptococcus* sp., and *Clostridium* spp., which validates other authors’ observations that this bacterial species is an effective biocontrol agent in aquaculture [18,79]. The EF supplemented with the analyzed probiotic isolate significantly improved the rainbow trout rearing parameters and apparent nutrient digestibility (protein, lipid, and NFE), and it contributed to the microbial safety of the juvenile rainbow trout. The *C. maltaromaticum* environmental probiotic strain effectively reduced and controlled the growth of pathogenic microorganisms, which is particularly important in the production of juvenile fish that are least resistant to the harmful effects of opportunistic and pathogenic microorganisms. The results of this study indicated that feed supplementation with the *C. maltaromaticum* environmental probiotic strain exerted a positive effect on juvenile rainbow trout, and they provide valuable inputs for future research into the isolate’s influence on other developmental stages of rainbow trout, as well as other species of farmed fish.

## 5. Conclusions

This study demonstrated that the *C. maltaromaticum* environmental probiotic strain is a biological growth stimulator that enhances the microbial safety of juvenile rainbow trout in RAS. During a 56-day rearing experiment, the EF (Aller Gold supplemented with 0.1% of *C. maltaromaticum*) significantly influenced (*p* < 0.05) rearing parameters, apparent nutrient digestibility (protein, lipids, ash, and NFE), as well as the counts of potentially pathogenic bacteria in the digestive tract contents and the skin of fish.

In fish receiving the EF with the addition of the probiotic isolate, final body weights (BW_f_) and body weight gains (BW_g_) increased by 14.44%; daily growth rate (DGR) increased by 25.34%; specific growth rate (SGR) increased by 13.77%; the viscerosomatic index (VSI) increased by 9.00%; and the final condition factor (K_f_) increased by 4.14% relative to fish fed with the CF. The apparent digestibility coefficients of protein, fat, ash, and NFE increased significantly by 3.29%, 1.00%, 1.01%, and 5.13%, respectively, in fish receiving the EF supplemented with 0.1% of *C. maltaromaticum*. The counts of potentially pathogenic bacteria (TCHMB, *P. fluorescens*, *A. hydrophila*, *Staphylococcus* spp., and *Clostridium* spp.) were also significantly reduced (10^2^–10^6^) in the digestive tract contents and the skin of juvenile rainbow trout, as well as in the EF.

The results of this study indicate that feed supplementation with the *C. maltaromaticum* environmental probiotic strain exerts a positive effect on juvenile rainbow trout, and they pave the way for future research into the isolate’s influence on other developmental stages of rainbow trout, as well as other species of farmed fish.

## Figures and Tables

**Table 1:** Microbiological assays and culturing conditions (temperature and incubation time) during analyses of the digestive tract contents, skin, and feed samples.

Assay	Culture Medium	Temperature and Incubation Time
Total counts of *Carnobacterium* *maltaromaticum* bacteria	Tryptone soy agar (TSA; Oxoid, Basingstoke, UK) with the addition of 3% yeast extract and 1.5% (*w*/*v*) NaCl [25]	28 °C/24 h
TCMB: total counts of mesophilic bacteria	Tryptone soy agar (TSA; Oxoid, Basingstoke, UK)	28 °C/48 h
TCHMB: total counts of hemolytic mesophilic bacteria	Tryptone soy agar (TSA; Oxoid, Basingstoke, UK) with 5% addition of defibrinated sheep blood	37 °C/48 h
Counts of *Pseudomonas fluorescens* bacteria	King B medium (Merck KgaA, Darmstadt, Germany)	28 °C/48 h
Counts of *Aeromonas hydrophila* bacteria	Aeromonas Medium Base (Ryan) (Merck KgaA, Darmstadt, Germany)	37 °C/24 h
Counts of *Staphylococcus* spp. bacteria	Chapman medium (Merck KgaA, Darmstadt, Germany)	37 °C/48 h
Counts of sulfite-reducing anaerobic spore-forming *Clostridium* sp. bacteria	Wilson-Blair medium (Merck KgaA, Darmstadt, Germany)	37 °C/18 h

**Table 2:** Rearing parameters and apparent nutrient digestibility in juvenile rainbow trout fed with a commercial feed (Aller Gold) and feed supplemented with 0.1% of the *Carnobacterium maltaromaticum* environmental probiotic strain (mean value ± SD; *n* = 3). The dietary treatments are described in the Materials and Methods section. Groups marked with different letters in the same row differ significantly (Mann–Whitney U test *p* < 0.05; *n* = 90).

Parameter	Juvenile Rainbow Trout Feeding	
	CF ^1^	EF ^2^
Initial body weight: BW_i_ (g)	44.20 ± 7.73 ^a^	44.40 ± 6.37 ^a^
Final body weight: BW_f_ (g)	89.17 ± 11.80 ^a^	104.10 ± 12.45 ^b^
Weight gain: BW_g_ (g juveniles^−1^)	44.97 ± 12.98 ^a^	59.70 ± 11.65 ^b^
Initial biomass: B_i_ (kg m^−3^)	5.30 ± 0.93 ^a^	5.33 ± 0.76 ^a^
Final biomass: B_f_ (kg m^−3^)	10.70 ± 1.42 ^a^	12.49 ± 1.49 ^b^
Biomass gain: B_g_ (kg m^−3^)	5.40 ± 1.56 ^a^	7.16 ± 1.40 ^b^
Daily growth rate: DGR (g day^−1^)	0.80 ± 0.23 ^a^	1.07 ± 0.21 ^b^
Initial condition factor: K_i_ (%)	1.18 ± 0.31 ^a^	1.19 ± 0.30 ^a^
Final condition factor: K_f_ (%)	1.19 ± 0.07 ^a^	1.24 ± 0.12 ^b^
Coefficient of variation of initial body weight: CVBW_i_ (%)	18.09 ± 3.53 ^a^	14.68 ± 2.42 ^b^
Coefficient of variation of final body weight: CVBW_f_ (%)	13.50 ± 2.05 ^a^	12.15 ± 1.64 ^b^
CVBW = CVBW_f_/CVBW_i_	1.34 ± 0.16 ^a^	1.20 ± 0.13 ^b^
Feed conversion ratio: FCR	1.06 ± 0.03 ^a^	0.89 ± 0.16 ^b^
Specific growth rate: SGR (% day^−1^)	1.19 ± 0.04 ^a^	1.38 ± 0.08 ^b^
Hepatosomatic index: HSI (%)	1.17 ± 2.10 ^a^	1.20 ± 0.18 ^a^
Viscerosomatic index: VSI (%)	14.56 ± 2.76 ^a^	16.00 ± 2.00 ^b^
Apparent digestibility coefficient of crude protein: PAD (%)	93.52 ± 0.49 ^a^	96.70 ± 0.20 ^b^
Apparent digestibility coefficient of crude fat: LAD (%)	95.01 ± 0.26 ^a^	95.97 ± 0.67 ^b^
Apparent digestibility coefficient of crude ash: AAD (%)	80.02 ± 0.10 ^a^	80.84 ± 0.19 ^b^
Apparent digestibility coefficient of nitrogen-free extract: NFEAD (%)	50.53 ± 0.48 ^a^	53.26 ± 0.43 ^b^

^1^ commercial feed (Aller Gold); ^2^ experimental feed (Aller Gold with 0.1% of *C. maltaromaticum*).

**Table 3:** Bacterial counts (mean ± SD, *n* = 14) in juvenile rainbow trout administered commercial feed (CF) (Aller Gold) and experimental feed (EF) (Aller Gold supplemented with 0.1% of the *Carnobacterium* environmental probiotic strain). The numbers marked with different superscript letters differ significantly (ANOVA; *p* < 0.05; *n* = 14).

Bacteria	Bacterial Counts (Mean ± SD) in Feed (cfu g^−1^)	
	CF ^1^	EF ^2^
*C. maltaromaticum*	n. f. ^3^	6.5 × 10^8^ ± 4.9 × 10^7 b^
TCMB ^4^	2.8 × 10^10^ ± 1.3 × 10^10 a^	9.6 × 10^5^ ± 4.9 × 10^5 b^
TCHMB ^5^	5.3 × 10^7^ ± 3.4 × 10^7 a^	1.6 × 10^2^ ± 0.9 × 10^2 b^
*P. fluorescens*	4.0 × 10^7^ ± 2.4 × 10^7 a^	1.9 × 10^2^ ± 2.4 × 10^1 b^
*A. hydrophila*	3.6 × 10^7^ ± 2.4 × 10^7 a^	8.2 × 10^2^ ± 2.1 × 10^2 b^
*Staphylococcus* spp.	4.8 × 10^7^ ± 1.3 × 10^7 a^	2.0 × 10^1^ ± 1.0 × 10^1 b^
*Clostridium* spp.	1.7 × 10^7^ ± 1.3 × 10^7 a^	3.0 × 10^1^ ± 1.0 × 10^1 b^

^1^ commercial feed; ^2^ experimental feed; ^3^ not found; ^4^ total counts of mesophilic bacteria; ^5^ total counts of hemolytic mesophilic bacteria.

**Table 4:** Correlation coefficients denoting the strength of the relationships between *Carnobacterium maltaromaticum* counts in the experimental feed, digestive tract, and skin samples (*n* = 44).

Sample	Bacteria	*C. maltaromaticum* Counts in		
		Experimental Feed	Digestive Tract	Skin
**Digestive tract**	*C. maltaromaticum*	0.7327 ***	n. d. ^3^	n. d.
	TCMB ^1^	−0.1139	−0.7678 ***	n. d.
	TCHMB ^2^	−0.9525 ***	−0.8726 ***	n. d.
	*P. fluorescens*	−0.7390 ***	−0.7256 ***	n. d.
	*A. hydrophila*	−0.9387 ***	−0.9109 ***	n. d.
	*Staphylococcus* spp.	−0.2620 ***	−0.9437 ***	n. d.
	*Clostridium* sp.	−0.7017 ***	−0.7678 ***	n. d.
**Skin**	*C. maltaromaticum*	0.3241 *	n. d.	n. d.
	TCMB ^1^	−0.1045	n. d.	−0.3648 *
	TCHMB ^2^	−0.3525 *	n. d.	−0.9515 **
	*P. fluorescens*	−0.4561 *	n. d.	−0.9826 *
	*A. hydrophila*	−0.4876 *	n. d.	−0.9274 **
	*Staphylococcus* spp.	−0.1623	n. d.	−0.9753 *
	*Clostridium* sp.	−0.4215 *	n. d.	−0.8479 **

^1^ total counts of mesophilic bacteria; ^2^ total counts of hemolytic mesophilic bacteria; ^3^ not determined; * statistically significant correlations at *p* < 0.05; ** at *p* < 0.01; *** at *p* < 0.001.

## Data Availability

The data presented in this study are available on request from the corresponding author.

## Supplementary Materials

The following supporting information can be downloaded at: https://www.mdpi.com/article/10.3390/ani12233321/s1, Table S1: The identification of an environmental strain of *Carnobacterium maltaromaticum* bacteria based on 16S rDNA sequence analysis.
