# Supplementary material for: The Influence of Commercial Feed Supplemented with Carnobacterium maltaromaticum Environmental Probiotic Bacteria on the Rearing Parameters and Microbial Safety of Juvenile Rainbow Trout
Source: Animals (Basel). 2022 Nov 28;12(23):3321. doi: 10.3390/ani12233321 (PMC9741101; doi:10.3390/ani12233321)
Supplement: Supplementary file 1 [file animals-12-03321-s001.zip › animals-2055181-supplementary.pdf]

Table S1. The identification of an environmental strain of *Carnobacterium maltaromaticum* bacteria based on 16S rDNA sequence analysis.

Results of identification	16S rDNA genes sequence	E-value
Carnobacterium maltaromaticum	CGCACGAAGTTGAAGAGCTTGCTCTTTAACCAAGTGAGTGGCGGACGGGTGAGTA<br>ACACGTGGGTAACCTGCCCATTAGAGGGGGATAACATTCGGAAACGGATGCTAAT<br>ACCGCATAGTTTCAGGAATCGCATGATTCTTCCAATGGAAAGGTGGCTTCGAAGCT<br>ACCACTAATGTTTGATGAGCACCACACGGCCCGCCACTAGCTATGTTCTGGGCCCT<br>TGCAGGTAAGTGGCGCACCAAGGCAATGATACGTAGCCGACCTGAGAGGGTGATC<br>GGCCACACTGGGACCGAAGTACATCCGCCCAGACTCCTACGGGAGGCTAGGCAGT<br>AGGGAATCTTACCGAAATGCACGAAAGTCTGAACCGGAGCAACTGCAACGCGTGA<br>GCGAACAAGGTTTACGGATCGTAAAACTCTGTTGTTAAAGAAGAACAAGCATCGA<br>GAGTAACAGCTCATTCCCCTGTACGGTACTAAACTGCAGAAAGCCACGGCTGGAA<br>CGACGTGCCAAACAGTGGCCGTCAGAGTAATACGTCACGGGTGGCAAGCGTTACG<br>ACCGTTGATCATTATTGGGCCCAAAGCGAGCAACAGCCCTGAATTCGGTTGTAGAG<br>TGCTGATGCTCGAAAGCCCCCGGCTCAACCGGGGAGGATCAGTGGAAACAGGAGA<br>ATCCCTTTCGAGATAGCAGATCTCACCAGGTTCCTACAGTTCTGGACCATTCCAAG<br>AGTAGCGCAACGAAATGCGTAGATATGGTGTACGAGGTGGCGAACACGCCCAGGG<br>GCAGTTAAGGAAACTCAAAGGTCGGTAACTGACGGCATGAGGGGTCCGCCTCACG<br>AATTACGCAATCGACGTCGTGACTTCCCGCCTTTGGGAGGTTGTGAATCGTGAGTA<br>GCAAATGCCGCACCTGATGGTCCCATAGATTAGGACAAACGAGGGGTGCGAAAGC<br>TCGGAGTCGCAGTCAATGTCTGGTCTCTCAGCGGAAGCGGTGACCACAAGGAGGT<br>GTATAGATGCGTAAAGTGGCGATGTGCACCTTAAGGTGAGAGGAGAAGACGTGAG<br>TTCAAGAGGTCAAAGGTTACTGGGAGGGGCCAACTCGGCCCCCGAAAGTGTAGTC<br>TGAATTTCTTGGCGGACG	1.0
